# Long-lived rodents reveal signatures of positive selection in genes associated with lifespan

**DOI:** 10.1371/journal.pgen.1007272

**Published:** 2018-03-23

**Authors:** Arne Sahm, Martin Bens, Karol Szafranski, Susanne Holtze, Marco Groth, Matthias Görlach, Cornelis Calkhoven, Christine Müller, Matthias Schwab, Johann Kraus, Hans A. Kestler, Alessandro Cellerino, Hynek Burda, Thomas Hildebrandt, Philip Dammann, Matthias Platzer

**Affiliations:** 1 Leibniz Institute on Aging–Fritz Lipmann Institute, Jena, Germany; 2 Department of Reproduction Management, Leibniz Institute for Zoo and Wildlife Research, Berlin, Germany; 3 European Research Institute for the Biology of Ageing, University of Groningen, University Medical Centre Groningen, Groningen, The Netherlands; 4 Department of Neurology; Jena University Hospital-Friedrich Schiller University, Jena, Germany; 5 Institute of Medical Systems Biology, Ulm University, Ulm, Germany; 6 Laboratory of Biology Bio@SNS, Scuola Normale Superiore, Pisa, Italy; 7 Department of General Zoology, Faculty of Biology, University of Duisburg-Essen, Essen, Germany; 8 University Hospital, University of Duisburg-Essen, Essen, Germany; Stanford University School of Medicine, UNITED STATES

## Abstract

The genetics of lifespan determination is poorly understood. Most research has been done on short-lived animals and it is unclear if these insights can be transferred to long-lived mammals like humans. Some African mole-rats (*Bathyergidae*) have life expectancies that are multiple times higher than similar sized and phylogenetically closely related rodents. To gain new insights into genetic mechanisms determining mammalian lifespans, we obtained genomic and transcriptomic data from 17 rodent species and scanned eleven evolutionary branches associated with the evolution of enhanced longevity for positively selected genes (PSGs). Indicating relevance for aging, the set of 250 identified PSGs showed in liver of long-lived naked mole-rats and short-lived rats an expression pattern that fits the antagonistic pleiotropy theory of aging. Moreover, we found the PSGs to be enriched for genes known to be related to aging. Among these enrichments were “cellular respiration” and “metal ion homeostasis”, as well as functional terms associated with processes regulated by the mTOR pathway: translation, autophagy and inflammation. Remarkably, among PSGs are *RHEB*, a regulator of mTOR, and *IGF1*, both central components of aging-relevant pathways, as well as genes yet unknown to be aging-associated but representing convincing functional candidates, e.g. *RHEBL1*, *AMHR2*, *PSMG1* and *AGER*. Exemplary protein homology modeling suggests functional consequences for amino acid changes under positive selection. Therefore, we conclude that our results provide a meaningful resource for follow-up studies to mechanistically link identified genes and amino acids under positive selection to aging and lifespan determination.

## Author summary

As an adaption to different environments rodents have evolved a wide range of lifespans. While most rodents are short-lived, along several phylogenetic branches long-lived species evolved. This provided us a unique opportunity to search for genes that are associated with enhanced longevity in mammals. Towards this, we computationally compared gene sequences of exceptional long-lived rodent species (like the naked mole-rat and chinchilla) and short-lived rodents (like rat and mouse) and identified those which evolved exceptional fast. As natural selection acts in parallel on a multitude of phenotypes, only a subset of the identified genes is probably associated with enhanced longevity. Applying several tests, we ensured that the dataset is related to aging. We conclude that lifespan extension in rodents can be attributed to changes in their defense against free radicals, iron homeostasis as well as cellular respiration and translation as central parts of the growth program. This confirms aging theories assuming a tradeoff between fast growth and long lifespan. Moreover, our study offers a meaningful resource of targets, i.e. genes and specific positions therein, for functional follow-up studies on their potential roles in the determination of lifespan–regardless whether they are currently known to be aging-related or not.

## Introduction

Most of the available information about the genetic mechanisms that govern lifespan and aging were obtained by studying single-gene mutations in invertebrates or short-lived, highly inbred vertebrate species. However, it is not clear whether insights about aging relevant genes and pathways gained from these species can be applied to long-lived species like human [1]. In addition, lifespan extensions under artificial laboratory conditions resulting from single gene mutations or other genetic, pharmacologic and/or lifestyle interventions are far smaller than natural variation of lifespan among species shaped by natural selection. Maximum lifespan of vertebrates varies about two orders of magnitude and is positively correlated with body mass [2, 3]. Therefore, comparative evolutionary approaches that search for genetic differences between closely related species that are long- and short-lived with respect to their body mass may reveal novel candidate genes and pathways or open new perspectives on known ones, e.g. by identifying amino acid sites under positive selection that are of potential functional relevance.

Rodents are an ideal taxon for such an approach. While the majority of species is short-lived, such as mice, rats and hamsters, there are long-lived exceptions, such as chinchillas, blind mole rats (*Spalax* sp.) and several African mole-rat species including the naked mole-rat (*Heterocephalus glaber*) [4, 5]. Furthermore, genome and transcriptome sequences of long- and short-lived species are available and can be used for comparative analysis.

**Fig 1:**
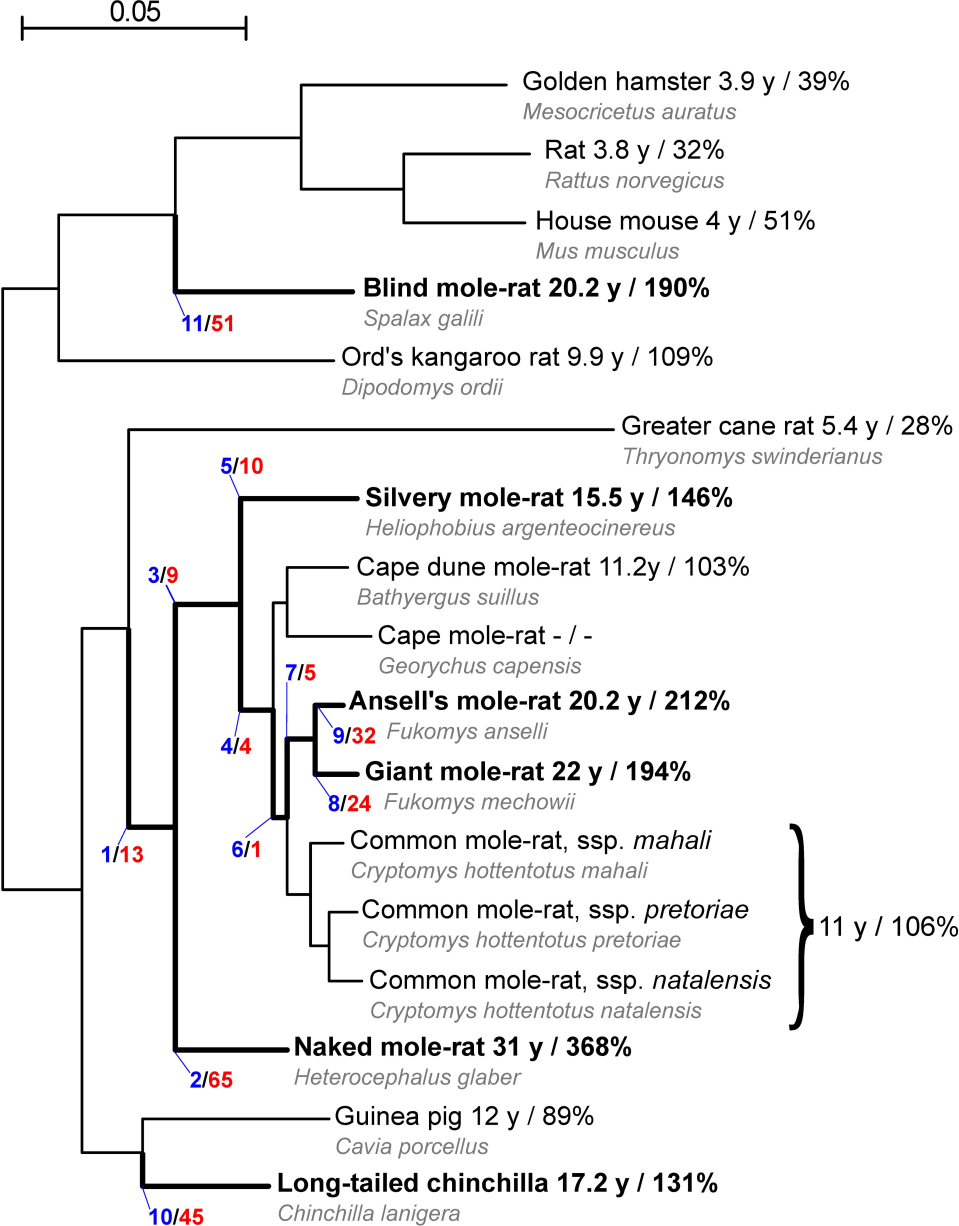
Nucleotide-based phylogeny of the analyzed rodents. Species or branches regarded in the present analyses as long-lived or leading to enhanced longevity, respectively, are depicted in bold. The branch numbers used in the text are shown in blue. The numbers of genes with signs of positive selection on the branches are colored in red. The first number after the species name shows the recorded maximum lifespan and the second number is the percentage of the observed vs. expected maximum lifespan based on the respective body mass. The maximum lifespans and ratios were taken from [4], except for silvery mole-rat (personal communication by R. Sumbera) and giant mole-rat (own data). For these two species, the expected maximum lifespans were calculated with the same mammalian allometric equation used by [4]. The scale bar represents 0.05 substitutions per site.

African mole-rats (family Bathyergidae) are subterranean rodents that feed from roots and tubers. The family comprises six genera; for five out of these, maximum lifespan records are available for at least one species. Notably, and in contrast to most other rodents, all of these species have a maximum lifespan of above ten years and exceeding the predictions of the power-law that describes body mass/lifespan relationships in mammals [5]. At the extreme of this distribution, Zambian mole-rats from the *Fukomys micklemi* clade [6] with its best studied representative Ansell´s mole-rat (*F*. *anselli*) and the giant mole-rat (*F*.*mechowii*), as well as naked mole-rat, have maximum lifespans of at least ca. 20, 22 and 31 years, respectively. These values are 212%, 194% and 368% with respect to the predicted lifespan based on their body mass ([4], giant mole-rat percentage calculated with own lifespan data and same formula). In contrast, the established biomedical model organisms mouse (*Mus musculus*) and rat (*Rattus norvegicus*) have a maximum lifespan of 3.8 and 4 years, respectively, which is 51% and 32% of the predicted value. Remarkably, the greater cane rat (*Thryonomys swinderianus*) that is closely related to the African mole-rats reaches only 28% of the predicted maximum lifespan (Fig 1).

Due to a number of unique phenotypes, the naked mole-rat became the focus of intensive research [7]. The naked mole-rat shows (i) the longest lifespan among rodents, (ii) minimal aging-related decline in reproductive and physiological parameters, as well as (iii) and an extremely low aging-related increase in mortality rate [8, 9]. Among thousands of examined animals only six recently discovered cases of spontaneous tumors have been described [10, 11]. Interestingly, cancer resistance is shared with blind mole rat, which is, despite its name, rather distantly related to African mole-rats (Fig 1). However, different mechanisms are proposed for cancer resistance in these two taxa.

The search for signatures of positive selection represents a powerful approach to identify the genetic basis of phenotypes of interest. Positive selection is the fixation of an allele in a taxon driven by its positive effect on fitness. It has, however, to be taken into account that selection/adaptation–in particular over long evolutionary time scales–is driven by a multitude of environmental stimuli that affect a multitude of phenotypes. Therefore, a genotype-phenotype link cannot be provided by such analysis. It requires additional knowledge and, finally, experimental validation. Respectively, bioinformatic positive selection studies are primarily hypothesis-driven and hypothesis-generating research. In protein-coding sequences (CDSs), positive selection results in an increased rate of non-synonymous substitutions as compared to genetic drift. Statistical models based on the ratio of non-synonymous to synonymous substitution rates (*dN*/*dS*) are widely used in comparative genomics and allow the identification of specific amino acids within a given gene that changed due to positive selection [12–14].

Consequently, several studies performed genome-scale scans for positively selected genes (PSGs) in African mole-rats and blind mole rat. The first study [15] searched for PSGs on the very long naked mole-rat branch in a four-species comparison with human as an outgroup and the mouse and rat as further rodents. Among the 142 identified PSG candidates, three were members of a five-protein complex involved in alternative lengthening of the telomeres. The second study [16], used ten species with the guinea pig (*Cavia porcellus*) as most closely related species and scanned for PSGs along the branches leading to naked mole-rat, Damaraland mole-rat (*Fukomys damarensis*) and their last common ancestor (LCA), identifying 334, 179 and 82 candidates, respectively, including candidates associated with neurotransmission of pain in the naked mole-rat. A third study [17] used species from all six African mole-rat genera and searched the branch of the LCA of all African mole-rats that follows divergence from the guinea pig. Signs of positive selection were identified in 513 genes, including loci associated with tumorigenesis, aging, morphological development and sociality. All three studies suffer from a methodological limitation that is common in positive selection studies: in none of these, a closer related species than guinea pig was included. As guinea pig is not the closest relative of African mole-rats not expressing the phenotypes of interest, it cannot be excluded that fixation of detected signs of positive selection predates–and therefore could not contribute to–the evolution of these phenotypes [18]. A fourth study [19] examined the blind mole rat branch using the Chinese hamster (*Cricetulus griseus*) as the most closely related outgroup. Among the 48 PSG candidates, several were linked to necrosis, inflammation and cancer.

To better resolve the above-mentioned ambiguities and to achieve a higher resolution of positive selection in respect to the evolution of enhanced longevity along rodent phylogenetic branches, we analyzed genomic and transcriptomic data of 17 species–data from public sources and original data generated for this study. In particular, we generated genomic data for the greater cane rat as a key species absent from previous analysis and for the silvery mole-rat (*Heliophobius argenteocinereus*). We systematically scanned 11 evolutionary branches associated with the evolution of enhanced longevity (6 corresponding to extant species and 5 to ancestral branches). This approach enabled us to date more precisely the occurrence of signatures of positive selection on multiple evolutionary branches.

In addition, we recently observed that PSGs in short-lived and fast-growing killifish were significantly more often up- than down-regulated during aging [14]. This finding is consistent with the concept of antagonistic pleiotropy [20] suggesting that the same genes that are positively selected for fast growth and maturation at young age are drivers of aging at old age. The antagonistic pleiotropy hypothesis is well supported, e.g. by the fact that growth rate and lifespan are negatively correlated, both between species and within many species [5, 21]. Respectively, we generated RNA-seq data from young and old naked mole-rats and rats to analyze the regulation of PSGs during aging, as selection may act both on the sequences of proteins and on their pattern of expression [22].

We found the identified PSGs to be enriched for genes related to aging and to show an expression pattern fitting the antagonistic pleiotropy theory of aging. Moreover, PSGs are linked to functional terms relevant to aging, like cellular respiration, metal ion homeostasis, regulation by the mTOR pathway, inflammation and the antioxidant defense. We discuss the implications of our results on the current understanding and for future follow-up studies of the genetic basis of aging and lifespan.

## Results and discussion

To gain new insights into the genetic mechanisms determining the lifespan in mammals, we performed a comparative genomic and transcriptomic study among long- and short-lived rodents. By searching for signs of positive selection on phylogenetic braches associated with the evolution of enhanced longevity, we aimed to provide a set of target genes/sites for future follow-up approaches to explore mechanistically their putative link to aging and lifespan determination. As natural selection acts in parallel on a multitude of phenotypes, we are aware that only a subset of the targets evolved under selection for enhanced longevity. To ensure that the dataset nevertheless represents a meaningful resource for aging research, we postulated four criteria for evaluation: (i) the PSGs show expression patterns during aging that are compatible with established theories of aging, (ii) the gene set is enriched for genes known to be aging-related, (iii) the gene set contains functional candidates for being relevant for aging but have not yet been associated therewith, and (iv) protein homology modeling of known aging-related genes suggests functional consequences for amino acid changes under positive selection.

As starting points for our analysis, we generated CDS libraries for five rodent species (four African mole-rat species and greater cane rat) based on transcriptomic and genomic data (S1/S2 Tables). Together with publicly available rodent CDS catalogs (S1 Table), we obtained data for 17 species, including several additional African mole-rats, chinchilla, blind mole rat and short-lived outgroups like the guinea pig, mouse and rat (Fig 1). From these sequences, we predicted orthologs and best matching isoforms between the species, calculated alignments and applied the branch-site test of positive selection to multiple branches [23].

Based on the lifespans of the extant species, we regarded six extant as well as five ancestral branches as leading to enhanced longevity and examined them for positive selection (Fig 1). We limited our analyses to those branches and did not consider a comparison to branches leading to short-lived rodents by two reasons. First, short/normal lifespan is widespread among rodents, and having a short lifespan is most likely the ancestral state in rodents. It is therefore much easier to identify phylogenetic branches on which lifespan was prolonged, e.g. within the mole-rat clade, than to identify branches on which lifespan was reduced. Second, there is an ongoing discussion that the same genes/pathways may be involved in the evolution of both short and long lifespan [24, 25], it is currently impossible to *in silico* predict whether positive selection in short- and long-lived species modulates gene functions in opposite directions.

In total, we detected 259 PSGs (false discovery rate (FDR) <0.1, branch-site test). Nine genes were found on multiple branches (S3 Table), resulting in a non-redundant set of 250 PSGs (S4 Table, S5–S15 Tables). Signs of positive selection for the same gene on multiple branches indicate possible parallel evolution. Among those, we found *AMHR2* (anti-Mullerian hormone receptor type 2) to be positively selected both on branch 2 (naked mole-rat) and branch 11 (blind mole rat). While AMHR2 plays a role in male fetal development and in ovarian follicle development of the adult female [26], no function with regard to aging is described yet. However, the protein kinase domain of AMHR2 contains the greatest number of longevity-selected positions based on a regression analysis with 33 mammalian species [27]. This domain contains 3 of 8 and 2 of 3 positively selected sites on branch 2 (naked mole-rat) and branch 11 (blind mole rat), respectively.

As a number of sequence/alignment characteristics potentially leads to a higher sensitivity of the underlying test of positive selection and thus could bias subsequent enrichment analysis, we specifically tested whether the identified PSGs had higher taxon coverage (S1 Fig) or greater sequence lengths (S2 Fig) than all tested genes. Neither was the case. Furthermore, analyses of GC-composition showed no differences between PSGs and all tested genes (S3/S4 Fig).

### Different studies on positive selection in mole-rats show minor overlaps

First, we compared our list of PSGs with the PSGs detected in previous studies of positive selection in mole-rats (S16 Table). As observed before, [17] PSGs from different studies show no or small overlaps. This is not surprising because the branches examined in previous studies differed from the branches examined in this study, even though some of them are named similarly. For example, Kim et al. examined a “naked mole-rat branch” using the house mouse as closest related species [15]. In our study, the sister taxon to naked mole-rat is represented by other African mole-rats and the house mouse is used only as an outgroup (Fig 1). In a similar way, the analysis of the African mole-rat ancestor by previous studies [17, 19] differs from ours as we incorporated the greater cane rat as closest related short-lived species and used guinea pig as an outgroup. We therefore analyzed evolutionary processes on a shorter phylogenetic distance that closely matches the appearance of the phenotypes under investigation. In addition, there are methodological differences between the studies, e.g. regarding ortholog prediction or alignment filtering. Unfortunately, the contribution of these technical variables to the discrepancies cannot be assessed as the alignments used for the previous studies are not available and cannot be compared with those generated and provided in our study (Supplement Data). Those five genes that were, despite the mentioned limitations, detected also by previous studies on the naked mole-rat branch (*AMHR2*, *IMP4*, *MYBPHL*, *MPZL2*, *TACC2*; S16 Table) can be considered as showing particular strong signals of positive selection.

### Positive selection leading to enhanced longevity and age-related expression are linked

Next, we analyzed the regulation of PSGs during aging–as selection may act both on the sequences of proteins and on their pattern of expression [19]–to identify potential links between positive selection on the analyzed branches and genetic determinants of lifespan. In general, directionality analysis of gene regulation during aging is complicated by the fact that the direction itself is not informative, whether the respective gene function is either causing or counteracting aging. For example, up-regulation of a causative gene may accelerate aging and shorten lifespan while adaptive up-regulation to counteract aging phenotypes may extend longevity. Based on our findings that up-regulation of PSGs in short-lived species may cause aging [14], we hypothesized that selection for enhanced longevity is more compatible with attenuation of gene activity–either on the level of protein function or gene regulation–since avoiding damage is easier than improving repair.

Moreover, genetics of aging is highly complex in general, and we do not assume that enhanced longevity evolved along the analyzed branches in the same way. But a number of genes/pathways are shown to be consistently involved in aging of even very distantly related taxa from yeast to mammals [28]. In addition, there are accumulating data that this is even more the case the closer related the taxa are [29, 30]. On this basis we hypothesize that there is a considerable overlap between the genes/pathways that are involved in aging among analyzed branches.

**Fig 2:**
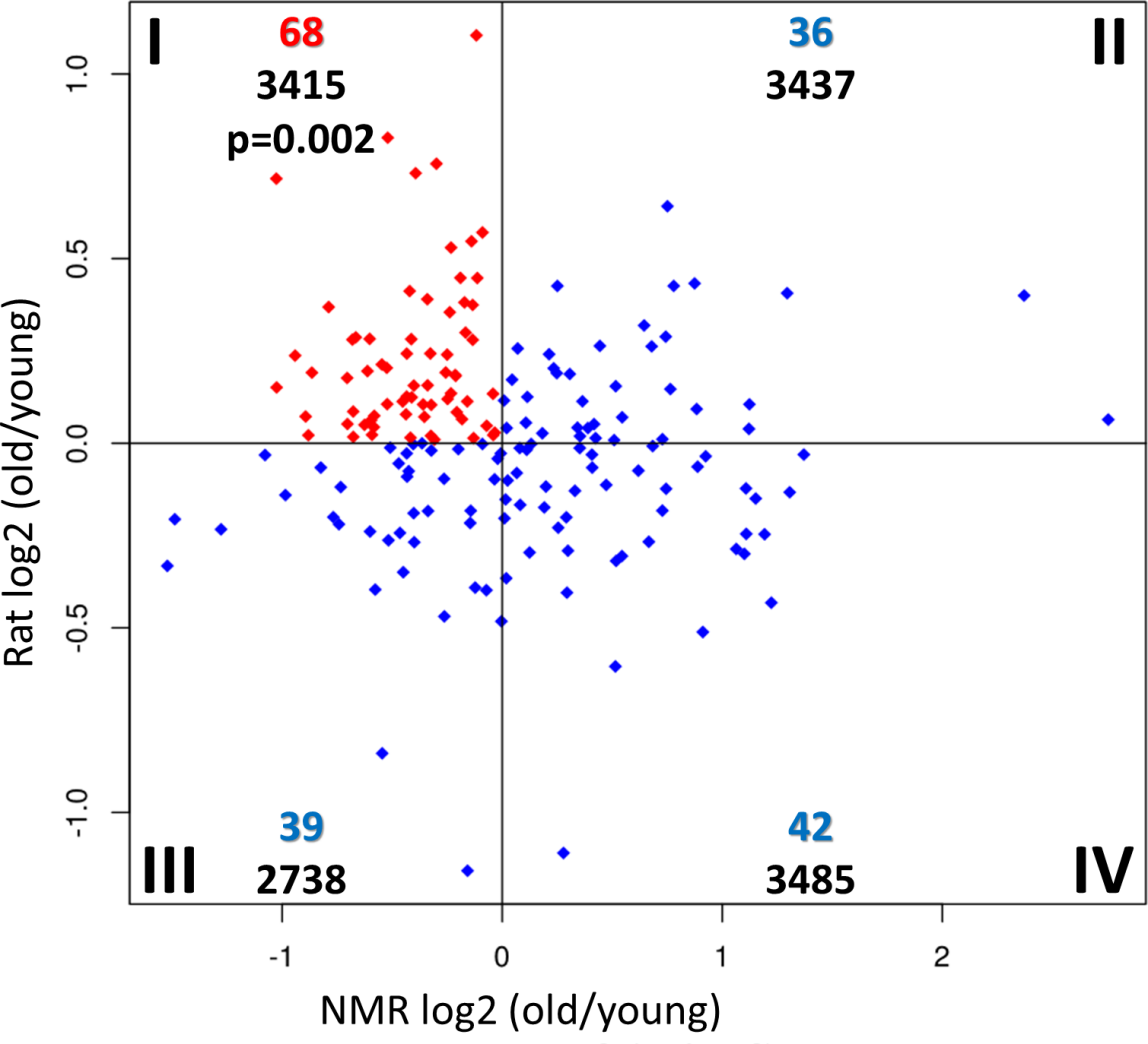
Changes of PSG expression in liver during aging of long-lived naked mole-rat and short-lived rat. The roman numbers describe the quadrant, the colored numbers below that show the number of PSGs in the respective quadrant and the black numbers at the bottom give the total regulated genes in the quadrant. The red marked quadrant (I) represents PSGs that were down regulated in naked mole-rat and up regulated in rat. This was tested against the sum of three blue marked quadrants (II, III, IV) with Fisher’s exact test (one-sided). The resulting p-value is shown in quadrant I. The total number of PSGs shown in this plot (185) is lower than the unique number of all PSGs (250) due to insufficient expression of genes (DEseq2) in at least one of the species.

To evaluate these hypotheses, we performed RNA-seq and subsequently compared gene expression in liver from old vs. young males of both long-lived naked mole-rats (>21 vs. 2–4 years) as well as short-lived rats (24 vs. 6 months; S17–S19 Tables). Indeed, the union set of PSGs across all examined branches showed during aging preference for down-regulation in naked mole-rat and for up-regulation in rats in respect to all regulated genes (p = 0.0029, Lancaster procedure [31]). Moreover, 68 PSGs were both down-regulated in the long-lived naked mole-rat and up-regulated in the short-lived rat during aging (Fig 2), resulting in a highly significant preference for quadrant I (down in naked mole-rat, up in rat; p = 0.0017, one-sided fisher test, quadrant I against the sum of II, III, IV). These results indicate that identified PSGs are associated with expression changes during aging of long- and short-lived rodents consistent with the antagonistic pleiotropy theory of aging.

### Positively selected genes are enriched for functional terms related to aging

To test whether the PSGs are enriched for genes known to be aging-related, we performed gene ontology (GO) term enrichment analysis. Among 16,639 naked mole-rat genes that were analyzed in total with regard to their expression, 2,220 genes were down-regulated and 3,212 genes up-regulated during naked mole-rat aging. Regarding all genes, there was a significant perturbation towards down-regulation during aging in 159 GO terms while 8 terms were perturbed towards up-regulation (FDR<0.1, GAGE; S20 Table). The enriched 159 terms were summarized into 7 categories (REVIGO, S21/S22 Tables). Among the three by far largest categories based on the number of covered genes are “cellular respiration” (GO:0045333, rank one), “translation” (GO:0006412, three) previously linked to aging (see below). While the other process “SRP-co-translational protein targeting to the membrane” (GO:0006614, two) may be related to aging as it is functionally connected to “translation”.

With respect to possible pleiotropic effects, translation and cellular respiration are also key components of the growth program. To evaluate the PSGs in respect to these categories, we built the union of genes for each REVIGO category and tested for overrepresentation of PSGs. Regarding all PSGs, there was a significant overlap with “cellular respiration” (p = 2.3*10^−4^, one-sided fisher test). Regarding only the 68 PSGs that were down-regulated in naked mole-rat and up-regulated during rat aging (quadrant I, Fig 2), “cellular respiration” showed a significant (p = 2.4*10^−7^) and “translation” a borderline significant enrichment (p = 0.10; S23 Table). This again suggests that PSGs are linked to aging relevant processes in an antagonistic pleiotropic way. The result is also consistent with the hyperfunction theory of aging that suggests that antagonistic pleiotropy works via a mechanism of “perverted” growth. According to this theory the growth program that is beneficial during youth is not entirely stopped after finishing development and causes damage from that point on. The theory further claims that the master regulator mTOR (mechanistic target of rapamycin) governs this growth program [32, 33].

### Regulation of mTOR- and downstream processes show signs of positive selection leading to enhanced longevity

The kinase mTOR operates as a central regulator of cell metabolism, growth, inflammation and proliferation. It was identified as a key regulator of aging and aging-related diseases in yeast, nematodes, fruit flies, and mice [34, 35]. On branch 2 (naked mole-rat), we found *RHEB* (Ras homolog enriched in brain) coding for the direct, positive regulator of mTOR and on branch 9 (Ansell’s mole-rat) its paralog *RHEBL1* to be positively selected, a situation consistent with the concepts of parallel evolution as well as of subfunctionalization of genes after duplication. mTOR can be activated by RHEB either on the surface of the peroxisome [36]–in response to reactive oxygen species (ROS)–or on the surface the lysosome [37]–in response to amino acids. We found peroxisomal genes (GO:0005777) enriched for PSGs both in the union across all examined branches (FDR = 0.026) as well as on branch 1 (LCA of the African mole-rats, FDR = 0.002). With regard to the lysosome, the major lysosomal membrane component *LAMP2* (lysosomal associated membrane protein 2), was identified as PSG on branch 11 (blind mole rat) and has a tendency towards positive selection on branch 2 (naked mole-rat, FDR = 0.11). LAMP2 is estimated to contribute together with its paralog LAMP1 about 50% of all lysosomal membrane proteins.

Despite that mTOR is partially regulated at the lysosome, it is also a key regulator of autophagy [38]. Autophagy is a cellular protective cleaning mechanism, required for organelle homeostasis, especially mitochondria. While enhanced autophagy was shown to be associated with lifespan extension in worms, flies and mice, inhibition of autophagy, conversely, leads to premature aging in mice [39]. LAMP2 (see above) acts also as a receptor for chaperone-mediated autophagy. It is required for degradation of individual proteins through direct import into the lysosomal lumen [40, 41]. Aging-dependent decrease of *LAMP2* expression was observed in mouse liver. Reinstatement of juvenile LAMP2 levels in aged mice significantly reduces aging-dependent decline of cell function and restores the degree of cell damage to that found in young mice [42].

Besides the lysosome, another cellular protein quality control and degradation system is the proteasome. While impaired proteasome function and subsequent accumulation of misfolded proteins were tightly correlated with aging and aging-related neurodegenerative disorders like Parkinson’s and Alzheimer’s disease, long-lived humans have sustained proteasome activity [43–45]. Two proteasome subunit genes, *PSMG1* (proteasome assembly chaperone 1) and *PSMB4* (proteasome subunit beta 4), were identified as PSGs on branch 11 (blind mole rat). PSMB4 has been classified as a driver for several types of tumors [46], is down-regulated during naked mole-rat aging (FDR = 0.088; DESeq2) and is a known interaction partner of PRP19 (pre-mRNA-processing factor 19 or senescence evasion factor) that is essential for cell survival and DNA repair [47].

Among mTOR-regulated processes that are relevant for both growth and aging are translation and cellular respiration [34]. Consistent with the observed antagonistic expression patterns of PSGs in the long-lived naked mole-rat and short-lived rat (see above), lower expression of genes related to these processes as well as pharmacological inhibition of the respective gene products was shown to be associated with longer lifespan in worms [48, 49], killifishes [50] and mice [51, 52].

Finally, mTOR is thought to play a critical role in regulating inflammatory and immune responses [53]. We found inflammatory response (GO:0006954; FDR = 0.027, Fisher’s exact test) and defense response (GO:0006952, FDR = 0.004) to be enriched for PSGs on branch 11 (chinchilla). Aging is tightly associated to the delicate balance between pro-inflammatory responses to resist potentially fatal infections and the inexorable damages that are accumulated by this [28, 54]. Chronic inflammation is described as a major risk factor for aging and aging-related diseases such as atherosclerosis, diabetes, Alzheimer's disease, sarcopenia and cancer [55].

IGF1 (insulin-like growth factor 1), a central regulator of the insulin/IGF1 pathway, was identified as PSG on branch 1 (LCA of African mole-rats). The insulin/IGF1 pathway is another aging relevant signaling pathway that links nutrient sensing to various anabolic and catabolic aspects of the metabolism [56]. Similar as for the mTOR-pathway, reduction of the insulin/IGF1 signaling increases longevity in worms, flies and mice and both pathways are linked by mutual feedback loops [34]. Furthermore, IGF1 is down-regulated during naked mole-rat aging (FDR = 1.6*10^−5^).

### Positive selection leading to enhanced longevity affects regulation of oxidative stress

With cellular respiration and peroxisome activity the main cellular sources of ROS were found to be affected by positive selection (see above). In small concentrations ROS can serve as signaling molecules, e.g. in regulation of mTOR [36] or apoptosis [57]. In higher doses, however, they can cause negative oxidative stress [58], i.e. damages to DNA, proteins and other cellular components [59]. Oxidative stress is thought to play a major role in the pathogenesis of neurodegenerative diseases [60] and even the determination of lifespan in general (“oxidative stress theory of aging”) [61]. On branch 3 (LCA of all African mole-rats except naked mole-rat), we found an enrichment of oxidoreductase activity (GO: GO:0016491; FDR = 0.056) and positive selection of *TXN* (thioredoxin), coding for an oxidoreductase enzyme that acts as an antioxidant extending lifespan in fly [62] and potentially also in mice [63, 64]. *SOD2* (superoxide dismutase 2) and *CCS* (copper chaperone for superoxide dismutase) are PSGs on branch 10 (chinchilla) and branch 2 (naked mole-rat), respectively. Both genes are involved in ROS defense and affect aging/lifespan in several species [65, 66]. This is interesting because in recent years, it has been repeatedly questioned that the oxidative stress theory of aging has much relevance for bathyergid rodents, given that several studies failed to find improved antioxidant capacities and/or less accumulation of oxidative damage in naked mole-rats compared to the much shorter-lived mice [67–69]. On the other hand, significantly higher levels of oxidative damage on proteins and lipids in non-reproductive as compared to reproductive females of the Damaraland mole-rat were found [70]. Since non-reproductive individuals live shorter (and hence age faster) than their reproductive counterparts in *Fukomys* sp. [71–73], these results are consistent with the oxidative stress theory of aging. The diverse signs of positive selection on branch 2 (naked mole-rat), 3 (LCA of all African mole-rats except naked mole-rat) and 7 (LCA of Ansell’s mole-rat and giant mole-rat) may suggest that the impact of oxidative stress on aging differs between naked mole-rat and other African mole-rats.

ROS production and ROS-induced damage to biomolecules are intertwined with the formation of advanced glycation end-products (AGEs). AGEs are stable bonds between carbohydrates and proteins/lipids which are formed in a non-enzymatic fashion. AGEs activate membrane-bound or soluble AGER (AGE specific receptor) and AGEs/AGER have been linked to several aging-related diseases including Alzheimer’s disease and diabetes [74]. Interestingly, *AGER* was found to be a PSG on branch 10 (chinchilla). The role of AGEs/AGER in aging is complex and ambivalent [75]. AGER is up-regulated in liver during naked mole-rat aging (FDR = 0.035). Similarly, in skin AGE levels rise with chronological age in Ansell’s mole-rat, but surprisingly are higher in the skin of slow aging breeders than of faster aging non-breeders [76]

### Positive selection leading to enhanced longevity affects metal ion homeostasis and transport

The majority of ROS is generated under participation of redox-active metals [77]. Therefore, disruptions of the metal ion homeostasis are thought to contribute to formation of free radicals. Furthermore, imbalances of metal ions, especially elevated iron levels, were associated with the pathogenesis of aging-related neurodegenerative diseases [78, 79]. Metal ion transport genes were found to be enriched for PSGs on branch 8 (giant mole-rat, GO:0030001, FDR = 0.025) and *TF* (transferrin) was identified as PSG on branch 4 (LCA of Cape, Cape dune, giant, Ansell’s mole-rat and common mole-rats). TF is an iron-binding protein responsible for transport of iron in the bloodstream and therefore essential for iron homeostasis [80]. Neurons regulate iron intake via the TF receptor and dysregulation of this tightly controlled process in the brain was shown to be highly associated with Parkinson’s and Alzheimer’s disease [81].

### Protein homology modeling suggests functional consequences of amino acid changes under positive selection

**Fig 3:**
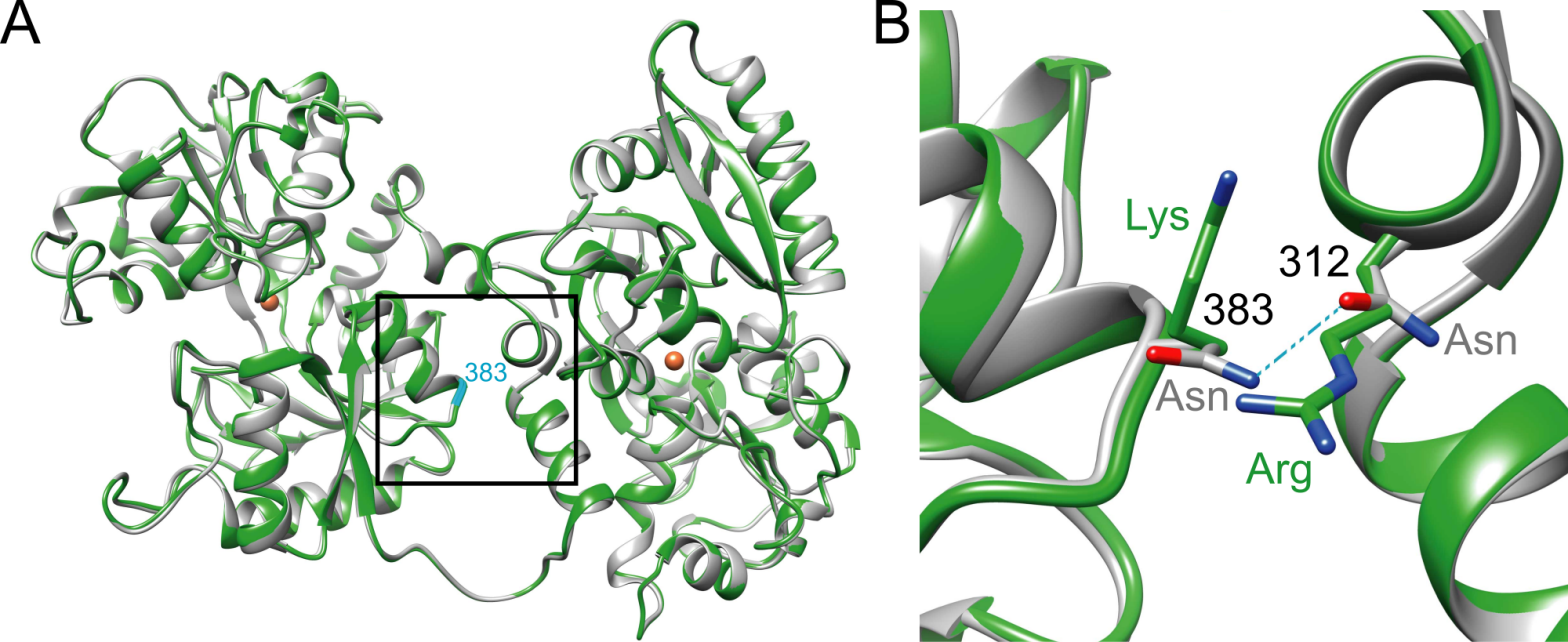
Homology models of Ansell’s mole-rat transferrin (TF). (A) Overview of the modeled Ansell’s mole-rat TF structure (green) superimposed onto the rabbit TF template structure (1JNF, grey). The position of the Asn383Lys site discussed in the text at the boxed center of the lobe interface numbered and indicated in cyan. Brown spheres: Fe^3+^ coordinated in the template structure (PDB ID: 1JNF; the radius of the metal ion is enlarged for better visibility). (B) Detail of the TF lobe interface. Shown is a magnification of the boxed region in (A). Coloring and numbering as in (A), side chain nitrogen atoms (blue), oxygen atoms (red). Potential hydrogen bond in 1JNF (light blue) as discussed in the text. Numbering (black) according to positions in the rabbit TF structure (1JNF).

Our positive selection analysis provides not only candidate genes but also candidate amino acids for functional follow-up studies. Protein homology modeling may reveal insights into the potential structural impact of a predicted positively selected amino acid change. As a proof of concept, we performed homology modeling for the site of highest probability of selection in TF–Ser383Lys. Serum TFs form a bilobal structure, and each lobe contains two dissimilar domains with a single iron-binding site. Inspecting the structure of the Ansell’s mole-rat TF modeled on the rabbit protein (PDP ID: 1JNF; [82]) as template, we realized that Lys 383 is located at the interface between the two lobes (Fig 3A). In the rabbit TF two juxtapositioned Asn residues at position 383 and 312 might form an H-bond and this constellation could stabilize the inter-lobe interactions (Fig 3B). In contrast, the juxtaposition of the positively charged side chains of Lys383 and a conserved Arg312 in the Ansell’s mole-rat structural model would be expected to weaken the lobe-lobe interaction due to electrostatic repulsion. The functional consequences for TF implied by this modeling require experimental investigations.

## Conclusions

We provided a systematic scan for PSGs on evolutionary branches of the African mole-rat family and other rodents leading to enhanced longevity that examine d considerably more extant and ancestral branches and provides a high resolution of positive selection than previous studies.

Analyzing the gene expression of PSGs, we found a highly significant pattern of down-regulation in the long-lived NMR and up-regulation in the short-lived rat, fitting the antagonistic pleiotropy theory of aging [83]. The PSGs and enriched functional terms cover many of the processes that are regulated by the mTOR pathway, e.g. translation, autophagy and cellular respiration. Furthermore, with RHEB and RHEB1L, direct regulators of mTOR [84], and with IGF1, a central component of insulin-signaling, were found to be under positive selection. In addition, we linked positive selection with inflammation and the antioxidant defense, processes known to be involved in regulation of lifespan.

By providing statistical evidences that the set of identified PSGs represents a meaningful resource for aging research, our results may encourage experimental follow-up studies–regardless whether the gene is currently linked to aging or determination of lifespan or not–since all sequences and alignments including the identified positively selected sites are accessible via supplement data.

## Methods

### Ethics statement

For tissue collection, rats were euthanized with CO2, whereas mole-rats were either anaesthetized by 3% isoflurane inhalation (naked mole-rats) or 6 mg/kg ketamine combined with 2.5 mg/kg xylazine (giant mole-rats; see Garcia et al. 2015, doi: 10.1111/vaa.12180) and then euthanized by surgical decapitation. Animal housing and tissue collection was compliant with national and state legislation (Berlin: breeding allowance #ZH 156; animal experimentation approval G 0221/12, Landesamt für Gesundheit und Soziales, Berlin; Essen: breeding allowances 32-2-1180-71/328 and 32-2-11-80-71/345, ethics/animal experimentation approval 84–02.04.2013/A164, Landesamt für Natur-, Umwelt- und Verbraucherschutz Nordrhein-Westfalen).

### CDS data

We examined nine African mole-rat species covering all six genera. Additionally, our analysis comprises eight outgroup species, including the long-lived blind mole rat and the chinchilla. mRNA sequences of seven distantly related outgroup species were obtained from RefSeq along with their CDS annotation (S1 Table). For the naked mole-rat we used a recently published *de novo* transcriptome assembly [85]. RNA-seq data for six mole-rat species was obtained from GenBank Sequence Read Archive, study SRP061925 [17]. The reads were assembled and annotated using FRAMA as described in [85].

For Ansell’s and giant mole-rat, purification of RNA from 13 and 17 tissues, respectively, was done using Qiagen RNeasy Mini Kit following the manufacturer's description. Novel RNA-seq was performed for both species as described in S2 Table. *De novo* transcriptome assemblies of the generated data were performed using FRAMA with human as reference species [85]. In cases in which multiple isoforms per gene were annotated based on the reference, all of them were used in subsequent analyses. The completeness of the assemblies, estimated using BUSCO [86], was 54–100% (S1 Table).

For the silvery mole-rat and the greater cane rat genome sequencing was performed to complement the transcriptome data. DNA was isolated from liver tissue of two female silvery mole-rats and a male greater cane rat using DNeasy Blood & Tissue (Qiagen). DNA was then converted to Illumina libraries and sequencing was done as given in S2 Table. Sequence reads were cleaned by removal of adaptors. Then, the reads were trimmed using the CLC quality trim program (parameters -l 0.5, -f 33 and -b 0.1), i.e. the largest region of each read was identified that that has at max. 10% of its bases with quality scores ≤20 and this region was used for further analysis only if it contained at least 50% of the total bases of the respective read (otherwise the read was discarded). Additionally, duplicons were discarded. *De novo* genome sequence assembly was performed using CLC assembler (Qiagen) with default settings. The CDS annotation was done using AUGUSTUS [87] with Ansell’s mole-rat CDSs as hint. The estimated completeness for these assemblies using BUSCO was 39% and 13% for silvery mole-rat and greater cane rat, respectively (S1 Table). For the greater cane rat, an additional, reference based coding sequence assembly was created by mapping greater cane rat reads against giant mole-rat transcripts using CLC mapper (-a local -l 0.4 -s 0.9) and introducing changes to the giant mole-rat transcripts using CLC variation finder (-z unknown -c 2 –I). The estimated completeness of this assembly was 91% (S1 Table).

All animals were housed and euthanized compliant with national and state regulations. Read data was deposited as ENA (European Nucleotide Archive) study PRJEB20584.

### Identification of positively selected genes

To scan on a genome-wide scale for genes under positive selection, we fed the CDSs of the described species set along with the branches we wanted to examine (Fig 1) into the PosiGene pipeline [88]. Giant mole-rat was used as PosiGene’s anchor species. Orthology was determined by PosiGene via best bidirectional BLAST searches [89, 90] against the orthologs groups defined in the HomoloGene [91] database (PosiGene parameter -hs for HomoloGene species and -nhs for species not included in the database). Regarding the silvery mole-rat, for which we had both a genome and a transcriptome assembly, we used generally the transcriptome assembly, except for those ortholog groups in which the silvery mole-rat ortholog could not be assigned via transcriptomic but via genomic data. This was accomplished by calling the three PosiGene modules separately, feeding both assemblies independently in the first module (ortholog assignment) and deleting all genome-based silvery mole-rat sequences in those ortholog groups that contained transcriptome-based silvery mole-rat CDSs before calling the second module. We considered all genes with Benjamini-Hochberg corrected p-values (FDR) < 0.1 as candidate genes. To exclude that these candidates were products of computational convergence problems that are known to potentially cause false positives in such analyses [92], we performed for each candidate two control runs with PosiGene. Only those candidates that were approved in both control runs were considered as PSGs and used in subsequent analyses (across all branches 8 candidates were removed, S5–S15 Tables).

The above described procedure was performed separately for the branches 2–11 and for branch 1 with different greater cane rat assemblies. While for the branches 2–11 the *de novo* greater cane rat assembly was used, for branch 1 the reference based greater cane rat assembly was used. The reason for this separation is the lack of completeness of the initial *de novo* greater cane rat assembly (S1 Table). The impact of this for branches 2–11 can assumed to be minimal as the greater cane rat is only one of nine potential outgroups. For branch 1 (LCA of African mole-rats), however, the greater cane rat is the sister taxon whose presence is required in an alignment to ensure that detected signs of positive selection do not predate branch 1 [18]. Therefore, to avoid a considerably lower sensitivity with respect to branch 1 than for other branches, the second, more complete, reference based greater cane rat assembly was used (S1 Table).

An overview about the number of genes and sequences tested for positive selection in the different branches is shown in S4 Table.

### Differentially expressed genes during naked mole-rat and rat aging

The young and old rats (strain Wistar) had an age of 6 (n = 4) and 24 (n = 5) months, respectively. The young naked mole-rats had an age of 3.42±0.58 years (average±sd, n = 6). The old naked mole-rats were at least 21 years old (recorded lifetime in captivity, n = 3). All examined animals were males. All animals were housed and euthanized compliant with national and state regulations. For both species, purification of RNA from liver samples was done using Qiagen RNeasy Mini Kit following the manufacturer's description. In short, we performed RNA-seq using Illumina HiSeq 2500 with 50 nt single read technology and a sequencing depth of at least 20 mio reads/sample (S17 Table). For naked mole-rat, the read mapping was performed with STAR [93] (—outFilterMismatchNoverLmax 0.06—outFilterMatchNminOverLread 0.9—outFilterMultimapNmax 1) against the public genome (Bioproject: PRJNA72441) that we had annotated before by aligning the above-mentioned naked mole-rat transcriptome reference using BLAT [94] and SPLIGN [95]. Rat reads were aligned against the mentioned RefSeq reference using bwa aln [96] (-n 2 -o 0 -e 0 -O 1000 -E 1000). Read data and counts were deposited as GEO (Gene Expression Omnibus) series GSE98746. Differentially expressed genes (FDR≤0.1, S18 and S19 Tables) and fold-changes were determined with DESeq2 [97]. GAGE [98] was used to determine enriched gene ontologies based on fold-changes (S20 Table). Gene ontologies with FDR≤0.1 were summarized using REVIGO (allowed similarity = 0.5) [99]. Four of the six largest summarized categories of the resulting treemap (S21/S22 Tables) were further analyzed due their aging relevance (representative terms given): “translation” (GO:0006412), “cellular respiration” (GO:0045333), “response to oxidative stress” (GO:0006979) and “iron ion homeostasis” (GO:0055072). For each of these categories the union of genes across gene ontology terms was built. These unions were tested for significant overlaps with (i) the union of PSGs across branches and (ii) the union of PSGs across branches that were down-regulated during aging in naked mole-rat and up-regulated in rat (Fisher’s exact test). Functional annotation of the PSGs in respect to the four categories is given in S23 Table).

### Gene ontologies

We determined enrichments for GO categories with Fisher’s exact test based on the R package GOstats (S24 Table). The resulting p-values were corrected using the Benjamini-Hochberg method [100]. We used throughout the manuscript 0.1 as significance threshold.

### Homology modeling of protein structure

Models were built in SWISS-MODEL (http://swissmodel.expasy.org;) [101, 102]. No further optimization was applied to the resulting models. Superimposition of the model and template structures and rendering was carried out using CHIMERA [103].

## Supporting information

S1 Fig Taxon coverage. (PDF)

S2 Fig Lengths of examined sequences. (PDF)

S3 Fig GC−content of examined sequence. (PDF)

S4 Fig Standard deviation of GC−content within alignments. (PDF)

S1 Table Data sources for assemblies and sequence statistics. (XLSX)

S2 Table Samples that were sequenced to create genome/transcriptome assemblies. (XLSX)

S3 Table PSGs on multiple branches. (XLSX)

S4 Table Overview of positively selected genes (FDR<0.1) on examined branches. (XLSX)

S5 Table Results on branch 1. (XLSX)

S6 Table Results on branch 2. (XLSX)

S7 Table Results on branch 3. (XLSX)

S8 Table Results on branch 4. (XLSX)

S9 Table Results on branch 5. (XLSX)

S10 Table Results on branch 6. (XLSX)

S11 Table Results on branch 7. (XLSX)

S12 Table Results on branch 8. (XLSX)

S13 Table Results on branch 9. (XLSX)

S14 Table Results on branch 10. (XLSX)

S15 Table Results on branch 11. (XLSX)

S16 Table Overlaps between this and previous studies. (XLSX)

S17 Table Samples that were RNA-sequenced to examine gene regulation during aging. (XLSX)

S18 Table DESeq2 result for gene expression comparison of young (Ø 3.42 years) vs. old (>21 years) naked mole-rats. (XLSX)

S19 Table DESeq2 result for gene expression comparison of young (6 months) vs. old (24 months) rats. (XLSX)

S20 Table GAGE gene ontology enrichment for expression changes during naked mole-rat aging (Ø 3.42 vs > 21 years, FDR<0.1). (XLSX)

S21 Table REVIGO treemap result of GAGE enrichment for differential expression during naked mole-rat aging. (XLSX)

S22 Table REVIGO representative categories (representative term given) of GAGE enrichment for differential expression during naked mole-rat aging. (XLSX)

S23 Table PSGs in aging relevant summarized REVIGO categories and quadrant 1 (up-regulated in rat and down-regulated in naked mole-rat). (XLSX)

S24 Table Gene ontologies enriched for PSGs on examined branches based on GOStats and Fisher's exact test (FDR<0.1). (XLSX)
