# Supplementary figures and images for: Long-lived rodents reveal signatures of positive selection in genes associated with lifespan
Source: PLoS Genet. 2018 Mar 23;14(3):e1007272. doi: 10.1371/journal.pgen.1007272 (PMC5884551; doi:10.1371/journal.pgen.1007272)

**S1 Fig. Taxon coverage.**

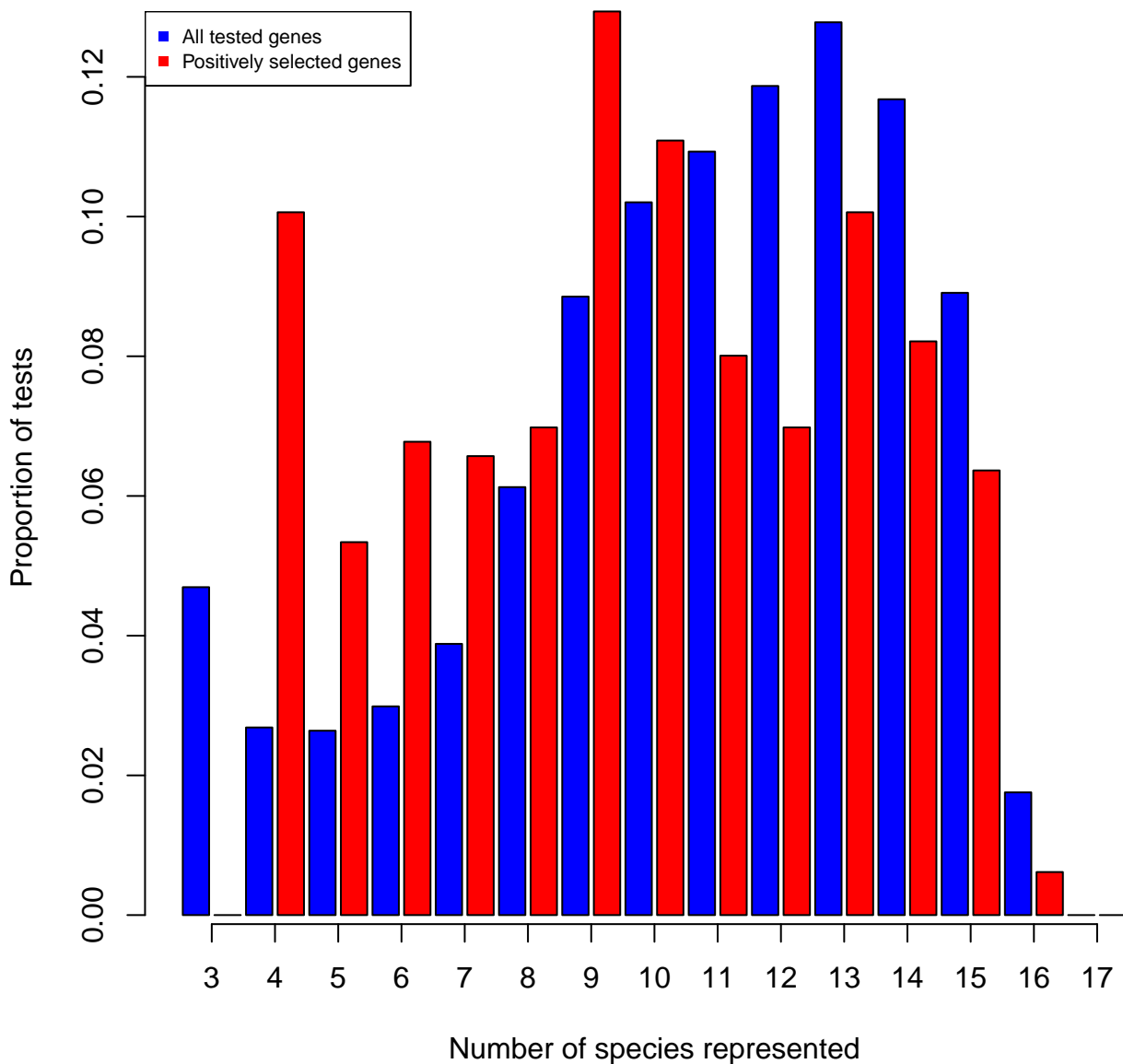

Supplement: S1 Fig — (PDF) [file pgen.1007272.s001.pdf]

**S2 Fig. Lengths of examined sequences.**

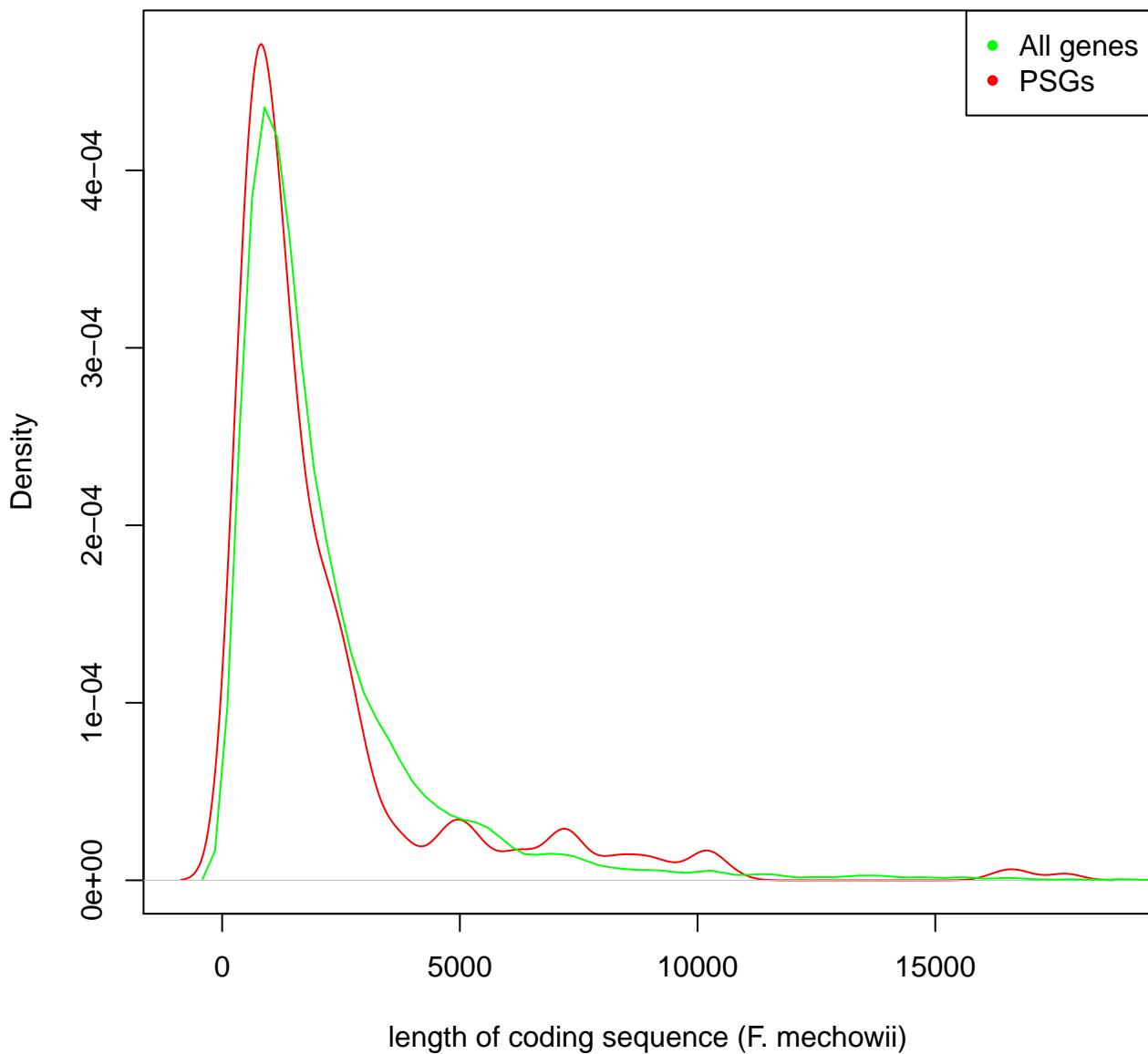

Supplement: S2 Fig — (PDF) [file pgen.1007272.s002.pdf]

**S3 Fig. GC-content of examined sequence.**

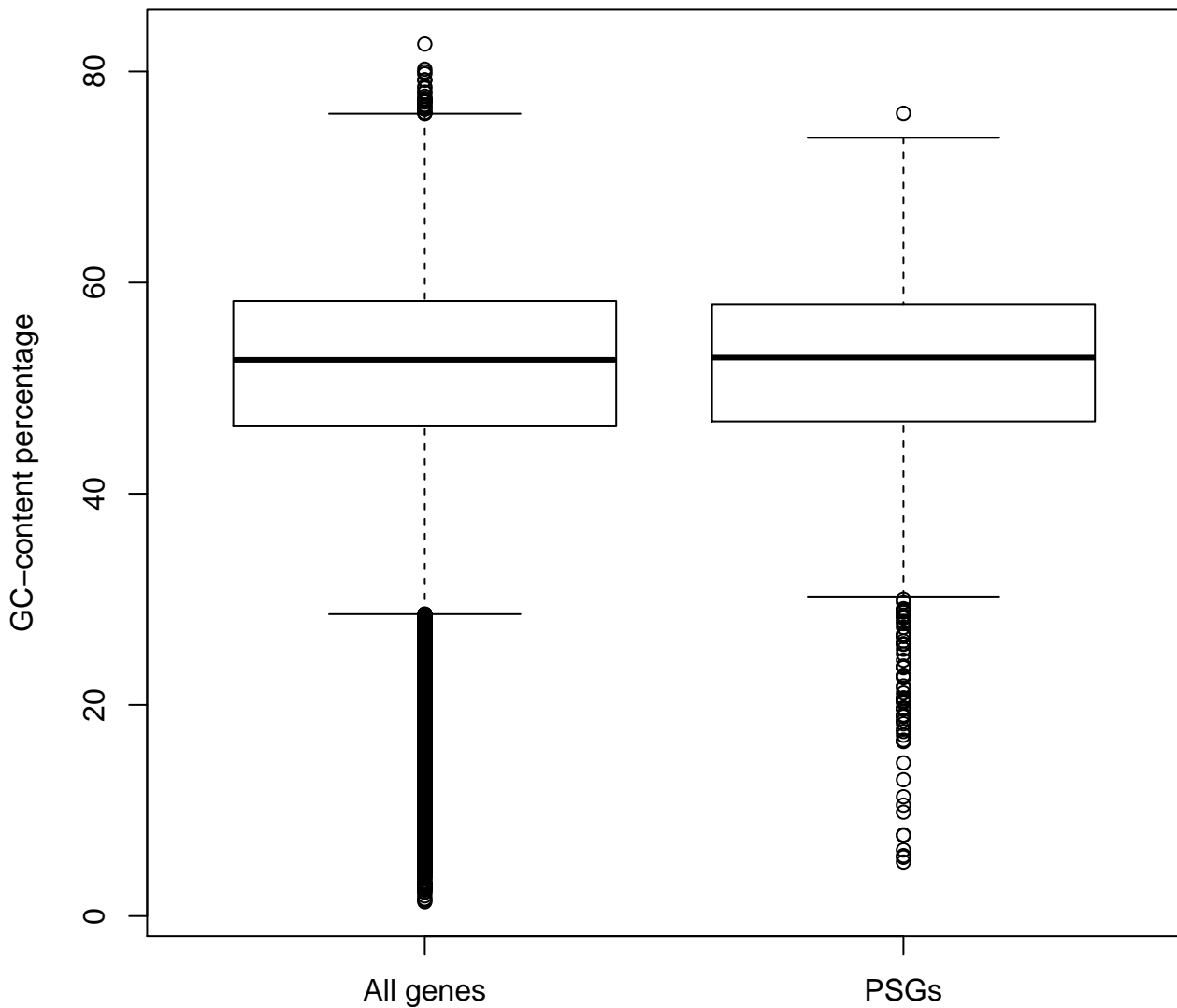

Supplement: S3 Fig — (PDF) [file pgen.1007272.s003.pdf]

**S4 Fig. Standard deviation of GC-content within alignments.**

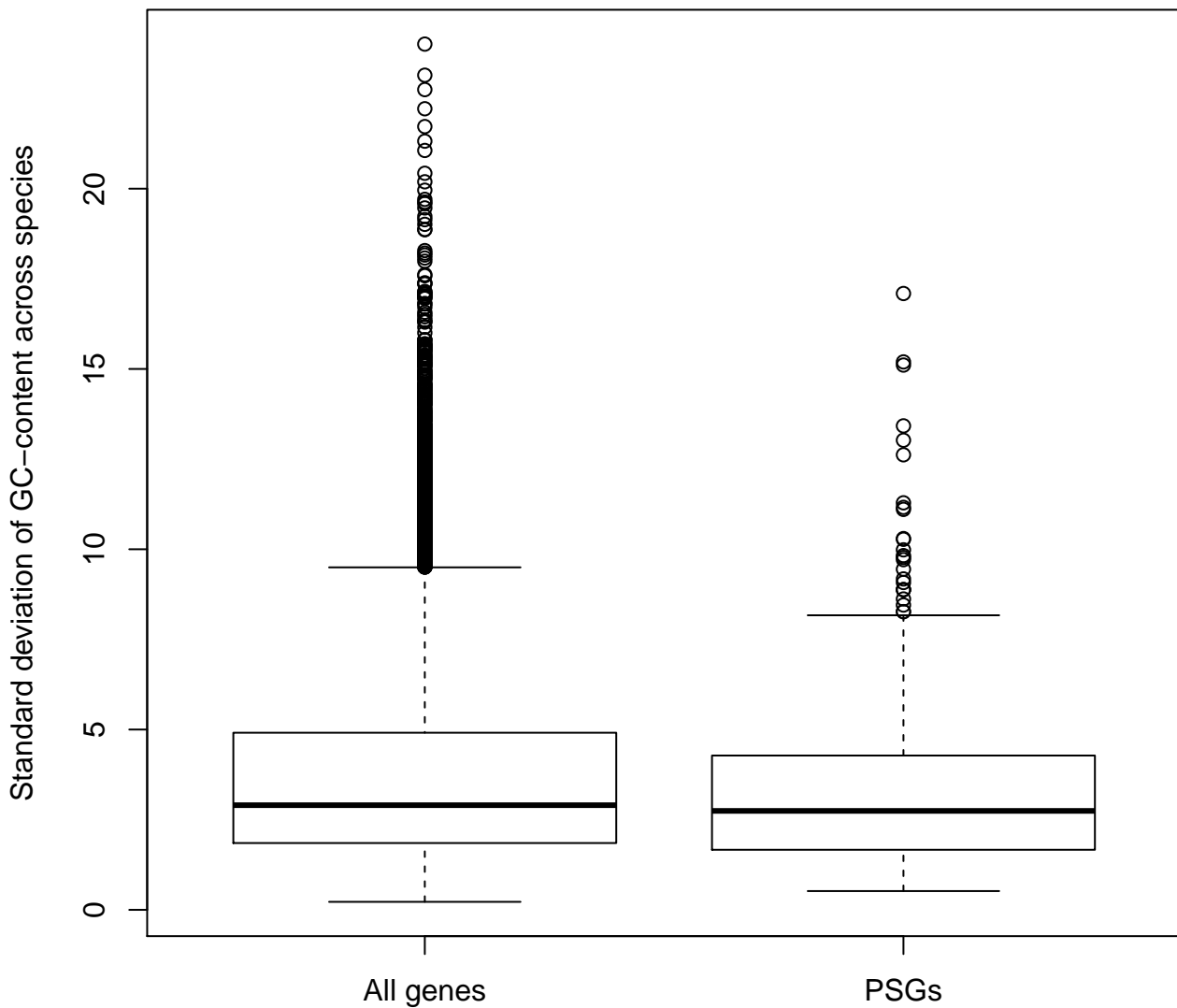

Supplement: S4 Fig — (PDF) [file pgen.1007272.s004.pdf]
